# Effects of dietary Hyssop, *Hyssopus officinalis*, extract on physiological and antioxidant responses of rainbow trout, *Oncorhynchus mykiss*, juveniles to thermal stress

**DOI:** 10.3389/fvets.2022.1042063

**Published:** 2022-10-20

**Authors:** Morteza Yousefi, Seyyed Morteza Hoseini, Evgeny Vladimirovich Kulikov, Sergey Borisovich Seleznev, Aleksandr Konstantinovich Petrov, Nikolay Valerievich Babichev, Margarita Vasilyevna Kochneva, Simon John Davies

**Affiliations:** ^1^Department of Veterinary Medicine, Peoples' Friendship University of Russia (RUDN University), Moscow, Russia; ^2^Inland Waters Aquatics Resources Research Center, Iranian Fisheries Sciences Research Institute, Agricultural Research, Education and Extension Organization, Gorgan, Iran; ^3^Agroengineering Department, Peoples' Friendship University of Russia (RUDN University), Moscow, Russia; ^4^Aquaculture Nutrition Research Unit (ANRU) Carna Research Station, Ryan Institute and College of Engineering, University of Galway, Galway, Ireland

**Keywords:** antioxidant, feed additive, glutathione, hepatic health, hormones

## Abstract

The present study aimed at assessing the effects of dietary Hyssop, *Hyssopus officinalis*, extract on rainbow trout, *Oncorhynchus mykiss*, responses to thermal stress. The juveniles (69.8 ± 0.38 g) were stocked in 12 through-flow tanks at a density of 12 fish per tank. Methanolic extract of Hyssop (HME) was added to diet at 0, 100, 250, and 500 mg/kg and the fish were fed (3% of biomass) over a 70-d period: 62 d at 13.3 ± 0.08°C and 7 d at 21–22°C. At the end of the trial, the plasma alanine aminotransferase (ALT), aspartate aminotransferase (AST), lactate dehydrogenase (LDH), triiodothyronine (T3), thyroxin (T4), cortisol, glucose, lactate, total antioxidant capacity (TAC), ascorbate, and the gill glutathione peroxidase (GPx), glutathione reductase (GR), glutathione-S-transferase (GST), and malondialdehyde (MDA). The results showed that HME had no significant effects on fish growth performance, survival, and feed efficiency. Dietary 250 mg/kg HME significantly decreased plasma ALT activity (*P* < 0.001), but showed no significant effects on plasma AST) (*P* = 0.106) activity, T3 (*P* = 0.992), and T4 (*P* = 0.070) levels. Thermal stress significantly (*P* < 0.001) increased plasma ALT and AST activities, but lowered plasma T3 and T4 levels. Dietary HME and thermal stress had interaction effects on plasma cortisol (*P* < 0.001), glucose (*P* = 0.007), lactate (*P* = 0.010), LDH (*P* = 0.005), TAC (*P* = 0.038), ascorbate (*P* < 0.001), and the gill GPx (*P* = 0.001), GR (*P* < 0.001), GST (*P* < 0.001), and MDA (*P* = 0.001). Thermal stress significantly increased plasma cortisol, glucose, lactate, and LDH, the gill GPX, GR, and GST, but dietary HME supplementation significantly reduced such elevations, particularly at 250 mg/kg level. Dietary HME significantly increased plasma TAC before the thermal stress and mitigated the stress-induced decreased in TAC, particularly at 250 mg/kg level. Dietary HME significantly decreased the gill MDA before and after the thermal stress, and lowest MDA was observed in 250 mg/kg HME level. Based on the present results, 250 mg/kg HME is recommended as suitable dose to improve antioxidative responses and hepatoprotection in rainbow trout under heat stress.

## Introduction

Global warming is a major concern in various aquaculture sectors, particularly in the culture of coldwater species (1). As a poikilothermic animal, fish are susceptible to water thermal fluctuations. Aquatic animals have diverse responses to thermal stress, depending on its nature, including impairment in oxygen consumption, metabolism, growth, reproduction, and osmoregulation that result in various tress responses, oxidative stress, and immunosuppression (2–4). Thermal stress has also been found to negatively affect fish hepatic health. Higher temperature induces oxidative stress in fish hepatic tissues that may damage hepatocytes and compromise their function (5, 6). Hepatic damage can be detected by circulating levels of alanine aminotransferase (ALT) and aspartate aminotransferase (AST) and it has been found that high temperatures induce elevation in blood ALT and AST in fish (6–8). Previous studies have demonstrated that different fish species such as armored catfish, *Hoplosternum littorale* (9), freshwater catfish, *Heteropneustes fossilis* (10), pikeperch, *Sander lucioperca* (11), common carp, *Cypinus carpio* (12), and black rockcod, *Notothenia coriiceps* (13) exhibit diverse responses to high water temperature including increase in antioxidant enzyme activities and lipid peroxidation.

According to the aforementioned facts, it is necessary to find techniques that suppress the adverse effects of thermal stress in fish. Rainbow trout, *Oncorhynchus mykiss*, is a coldwater fish and exhibits highest growth rate and health at a temperature range of 13–15°C and shows growth depression and health deterioration at temperatures above this range (14). A short-term (9 days) exposure to 22°C has induced growth retardation (15). Moreover, other studies have shown that elevated water temperature increases energy expenditure (16, 17), cell respiration (18), blood glucose and cortisol (19–21), and muscle and hepatic oxidative stress (21, 22) in rainbow trout, indicating the need for establishing protocols to reduce the adverse effects of thermal stress in farm raised trout.

Herbal feed additives are known for their wide beneficial effects such as anti-stress, antioxidant properties, immunostimulant capacity, and hepatoprotective characteristics. Thus, dietary herbal additives may be good candidate to suppress thermal stress in fish under intensive production conditions (23). There are few studies on this topic in the scientific literature; for example, Chinese rhubarb *Rheum officinale*, extract was tested by Liu et al. (24) on the growth and feed utilization performance and physiological and metabolic responses of giant freshwater prawn, *Macrobrachium rosenbergii*, under elevated temperature stress. More recently, dietary oregano essential oil on growth performance, intestinal integrity, immune competence and oxidative status of Nile tilapia, *Oreochromis niloticus*, subjected to acute heat stress was reported by Magouz et al. (25). *Laminaria* sp. meal Kamunde et al. (26), and lion's mane, *Hericium erinaceus*, meal Khieokhajonkhet et al. (27) as potent seaweed derived feed additives have been found to suppress physiological stress, oxidative stress, hepatic injuries, and inflammation in different species such as Atlantic salmon, *Salmo salar* and Nile tilapia. However, there is now a requirement to investigate potential benefits of other herbal materials in fish exposed to thermal stress to extend the current knowledge Hoseinifar et al. (28).

Hyssop, *Hyssopus officinalis*, is a popular medicinal plant distributed in the central and southern Europe and Middle East (29). The hyssop extract has antifungal, antibacterial, antiviral properties and widely used in food and cosmetics industry (30). Moreover, the Hyssop extract has presented radical-scavenging properties, *in vitro* (31). Nevertheless, there is no study evaluating the benefits of dietary supplementation of Hyssop in fish and shellfish. Accordingly, the aim of the present study was to investigate the potential benefits of dietary methanolic extract of Hyssop leaves (HME) in rainbow trout exposed to thermal stress, by evaluating stress and antioxidant responses and hepatic health.

## Materials and methods

### HME preparation

Locally available Hyssop flowers were acquired from a local store and exposed to a fan blower for 24-h for drying. The dried materials were powdered by a mill and mixed with methanol at a ratio of 1:10 (w:v). The mixture was kept at room temperature for 48 h with occasional mixing. The mixture was then filtered and the solution was dried at 37°C for 48 h. The residual was collected and preserved in capped bottle at 4°C. To explore the composition of the extract, 1 g of the dried extract was dissolved in methanol and used for GC-MS analysis (Supplementary material).

### Diets

In this study, four diets with different levels of HME (0, 100, 250, and 500 mg/kg) were used (Table 1). To make the diets, the components of the diet were first mixed, and then some water was added to the mixture to form a dough. The dough was turned to strands by extrusion using a meat grinder and after drying against a fan, they were crushed in appropriate sizes (3 mm). The chemical composition of the diets was determined based on the standard Association of Analytical Chemists methods (32).

**Table 1:** Diet ingredient composition and chemical composition of the experimental diets.

	**Dietary HME concentration (mg/kg)**			
Ingredients (g/kg)	0	100	250	500
Corn meal	50	49.9	49.75	49.5
Wheat meal	230	230	230	230
Soybean meal^a^	143	143	143	143
Soybean oil	36.3	36.3	36.3	36.3
Fish process by product^b^	120	120	120	120
Poultry by product^c^	400	400	400	400
Vitamin premix^d^	5	5	5	5
Mineral premix^e^	5	5	5	5
Methionine^f^	5.80	5.80	5.80	5.80
Lysine^g^	4.40	4.40	4.40	4.40
HME	0	0.10	0.25	0.50
**Proximate composition**				
Moisture	91.2	89.6	89.0	90.0
Crude protein	395	399	393	398
Crude fat	152	155	156	150
Crude ash	76.5	75.6	75.0	76.9
Crude fiber	34.1	35.0	34.9	35.0

a Crude protein 45%.

b Crude protein 54%; crude fat 18%.

c Crude protein 54%; crude fat 22%.

d Amineh Gostar Co. (Tehran, Iran). The premix provided the following amounts of vitamin to the diets (per kg): A: 1,600 IU; D3: 500 IU; E: 20 mg; K: 24 mg; B3: 12 mg; B5: 40 mg; B2: 10 mg; B6: 5 mg; B1: 4 mg; H: 0.2 mg; B9: 2 mg; B12: 0.01 mg; C: 60 mg; Inositol: 50 mg.

e Amineh Gostar Co. (Tehran, Iran). The premix provided the following amounts of minerals to the diets (per kg): Se: 0.15 mg; Fe: 2.5 mg; Co: 0.04 mg; Mn: 5 mg; Iodate: 0.05 mg; Cu: 0.5 mg; Zn: 6 mg; Choline: 150 mg.

f Faravar Lysine Pars Co., Tehran, Iran.

g Evonik Co., Essen, Germany, 99% purity.

### Fish rearing and thermal stress

**Figure 1:**
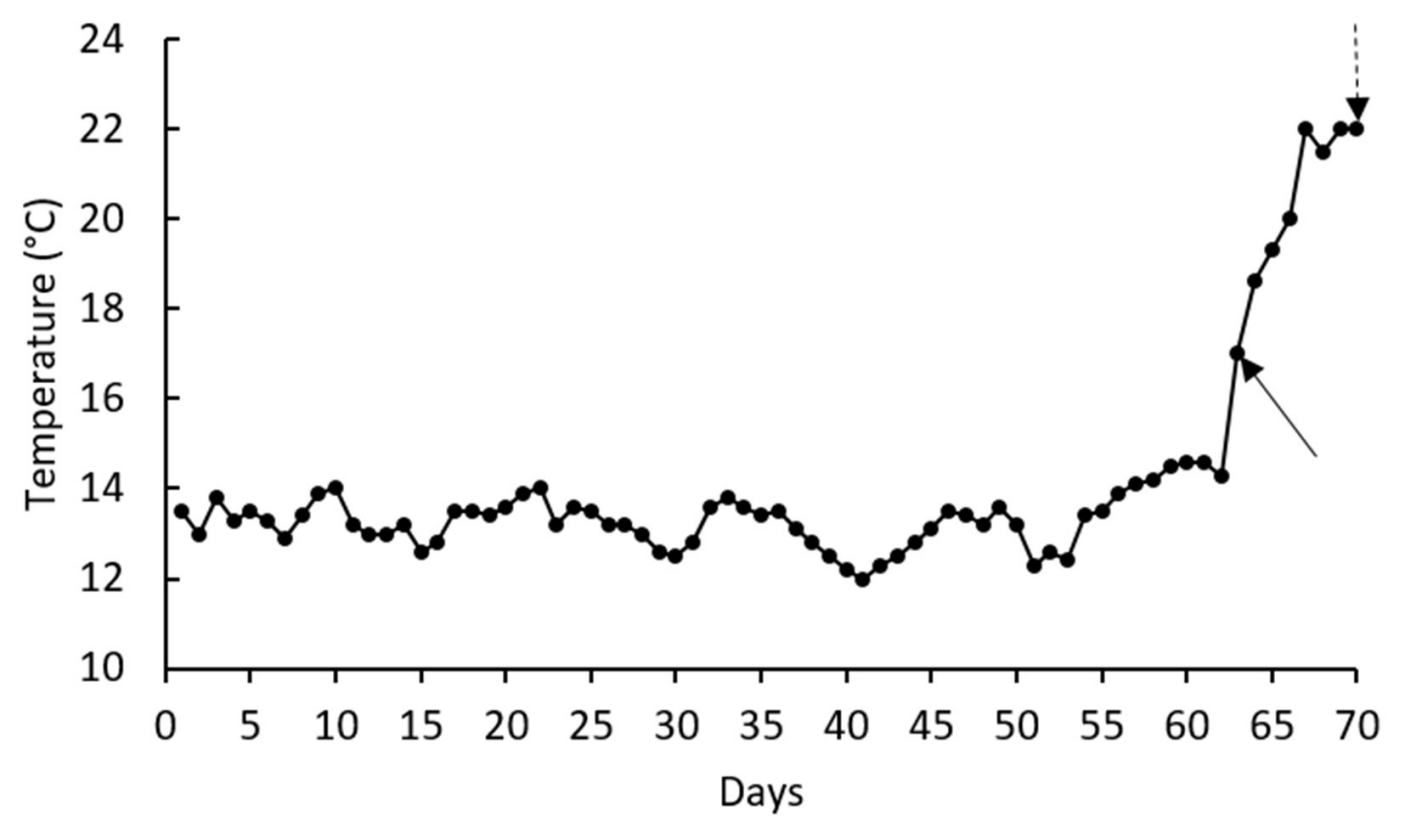
Mean daily water temperature during the 70-d rearing of rainbow trout. Solid arrow indicates the basal sampling day (water temperature = 16°C); whereas, dashed arrow shows stressed sampling day (water temperature = 22°C).

This study was conducted according to the guidelines of the Declaration of Helsinki and approved by the Peoples' Friendship University of Russia (RUDN University) ethical committee (EC1/351, 05/06/2021). Two hundred rainbow trout with an average weight of ~60 g were purchased from a private sector and transferred to the laboratory. After 1 week rearing in a holding tank (1,500 L), 144 healthy fish were randomly distributed into 12 tanks filled with 150 L water. Each of the aforementioned diet was offered at 3% of biomass to a batch of three tanks for 70 days. Water was supplied from a well and water flow rate in each tank was 0.3 L/kg.min. Water temperature was determined every day, whereas water pH (7.77 ± 0.31) and dissolved oxygen (8.55 ± 0.40 mg/L) levels were recorded twice a week (measured by portable probes provided by Hach Co. Colorado, USA). Water total ammonia levels (0.12 ± 0.02 mg/L) were recorded bi-weekly (measured by a photometer and kit provided by Palintest Co., Gateshead, UK). Mean water temperature during the first 62 days of rearing was 13.3 ± 0.08°C, but gradually increased up to 21–22°C during the days 67th−70th (Figure 1). The fish were sampled at the day 63rd (as basal point) and the day 70th (as thermal-stressed point). At the day 70th, the fish specific growth rate (SGR), feed conversion ratio (FCR), and weight gain were recorded as follow:


$$\begin{array}{r} SGR\left(\%/d\right)=100\times \frac{Ln\;\left(final\;weight\right)-Ln\;\left(initial\;weight\right)}{70}\\ FCR=\frac{Consumed\;feed\;\left(g\right)}{Gained\;biomass\;\left(g\right)}\\ Weightgain\left(\%\right)=100\times \frac{Final\;weight\;\left(g\right)-Initial\;weight\;\left(g\right)}{Initial\;weight\;\left(g\right)} \end{array}$$

### Sampling

At each sampling point (63rd and 70th days), two fish were captured from each tank and anesthetized in a eugenol bath (100 μL/L). Then, heparinized syringes were used to collect blood samples from the caudal vein. After blood sampling the fish were killed by a sharp blow on the head and sectioning of the spinal cord. The fish gill samples were collected by dissecting the right side gills (except the first arch).

The blood samples were centrifuged (3,000 g; 7 min; 4°C) to obtain plasma, which was stored at −70°C until analysis. The gill samples were immediately frozen in liquid nitrogen and transferred to laboratory. Then, the tissues were homogenized with 5 volume of cold phosphate buffer (pH 7.0). The homogenate was centrifuged (9,500 g; 15 min; 4°C) and the supernatant was recovered and stored at −70°C until analysis.

### Plasma analysis

Plasma cortisol levels were determined by a commercial ELISA kit (Monobind Co.; Lake Forest, CA 92630 USA). The kit was based on a competitive ELISA method with the detection range of 0–500 ng/mL. The kit sensitivity was 2.5 ng/mL with inter- and intra-assay variations of 7.32 and 4.65%, respectively.

Plasma T3 and T4 levels were determined using commercial competitive ELISA kits provided by Pishtazteeb Co. (Tehran, Iran). Detection range for T3 and T4 were 0–10 ng/mL and 0–20 μg/mL, respectively. Inter- and intra-assay variations for T3 were 5.62 and 3.22%, respectively. Inter- and intra-assay variations for T4 were 7.02 and 4.80%, respectively.

Plasma glucose (glucose oxidase method), lactate (lactate oxidase method), ALT (conversion of alanine to pyruvate), AST (conversion of aspartate to oxaloacetate), lactate dehydrogenase (LDH; reduction of NAD to NADH) were determined using Parsazmun Co. kits (Tehran, Iran) in an autoanalyzer (BS-480 Clinical Chemistry Analyzer, Shenzhen P. R. China).

Plasma total antioxidant capacity (TAC) was determined based on the oxidation/reduction method, using a commercial kit provided by Zellbio Co. (Deutschland, Germany). Detection range of the kit was 0.125–2 mM/L with a sensitivity of 0.1 mM/L. Plasma ascorbate level was measured based on the oxidation with 2,4- dinitrophenylhydrazine to produce a red color. The color intensity was proportional to the sample ascorbate level (33).

### Gill antioxidant parameter determination

The enzyme extracts were thawed and used for antioxidant assays. The gill glutathione peroxidase (GPx) and glutathione reductase (GR) activities were determined based on conversion of reduced glutathione to glutathione disulfide using a commercial kit (Zellbio Co.; Deutchland, Germany). The gill glutathione-S-transferase (GST) activity was measured based on dinitrophenyl-S-glutathione formation using a commercial kit (Zellbio Co.; Deutchland, Germany). The gill malondialdehyde (MDA) levels were determined based on the reaction with thiobarbituric acid at 95°C, after an initial de-proteinization using trichloroacetic acid and at the presence of butylated hydroxyl toluene (34). The samples soluble protein contents were determined according to the Bradford method (35).

### Statistical analysis

Normal distribution of the data and variance homogeneity were confirmed by Shapiro-Wilk's and Levene's tests, respectively. The growth data were subjected to one-way ANOVA and Duncan tests to find significant effects of dietary HME and significant differences among the treatments. The other data were subjected to two-way ANOVA, considering HME and thermal stress as factors. Significant differences among the treatments were checked by Duncan test. Significance was checked at *P* < 0.05 and the data were presented as mean ± SE. SPSS v.22 was used for analysis.

## Results

There were no mortalities during the rearing period and high temperature stress application. Dietary HME induced no significant changes in the fish final weight (*P* = 0.709), weight gain (*P* = 0.782), SGR (*P* = 0.787), and FCR (*P* = 0.623) after 70 days rearing (Table 2).

**Table 2:** Growth performance and feed efficiency of rainbow trout fed diets supplemented with 0–500 mg/kg HME over 70 days (mean ± SE; *n* = 3).

	**Dietary HME levels (mg/kg)**				
	**0**	**100**	**250**	**500**	**P value**
Initial weight (g)	69.9 ± 0.919	69.8 ± 0.719	69.8 ± 0.740	69.9 ± 1.12	0.953
Final weight (g)	247 ± 10.6	246 ± 12.4	251 ± 7.15	237 ± 3.30	0.709
Weight gain (%)	254 ± 18.4	253 ± 21.1	260 ± 7.35	239 ± 7.41	0.782
Specific growth rate (%/d)	1.80 ± 0.07	1.80 ± 0.09	1.83 ± 0.03	1.74 ± 0.03	0.787
Feed conversion ratio	1.42 ± 0.07	1.42 ± 0.07	1.39 ± 0.02	1.49 ± 0.03	0.623

**Figure 2:**
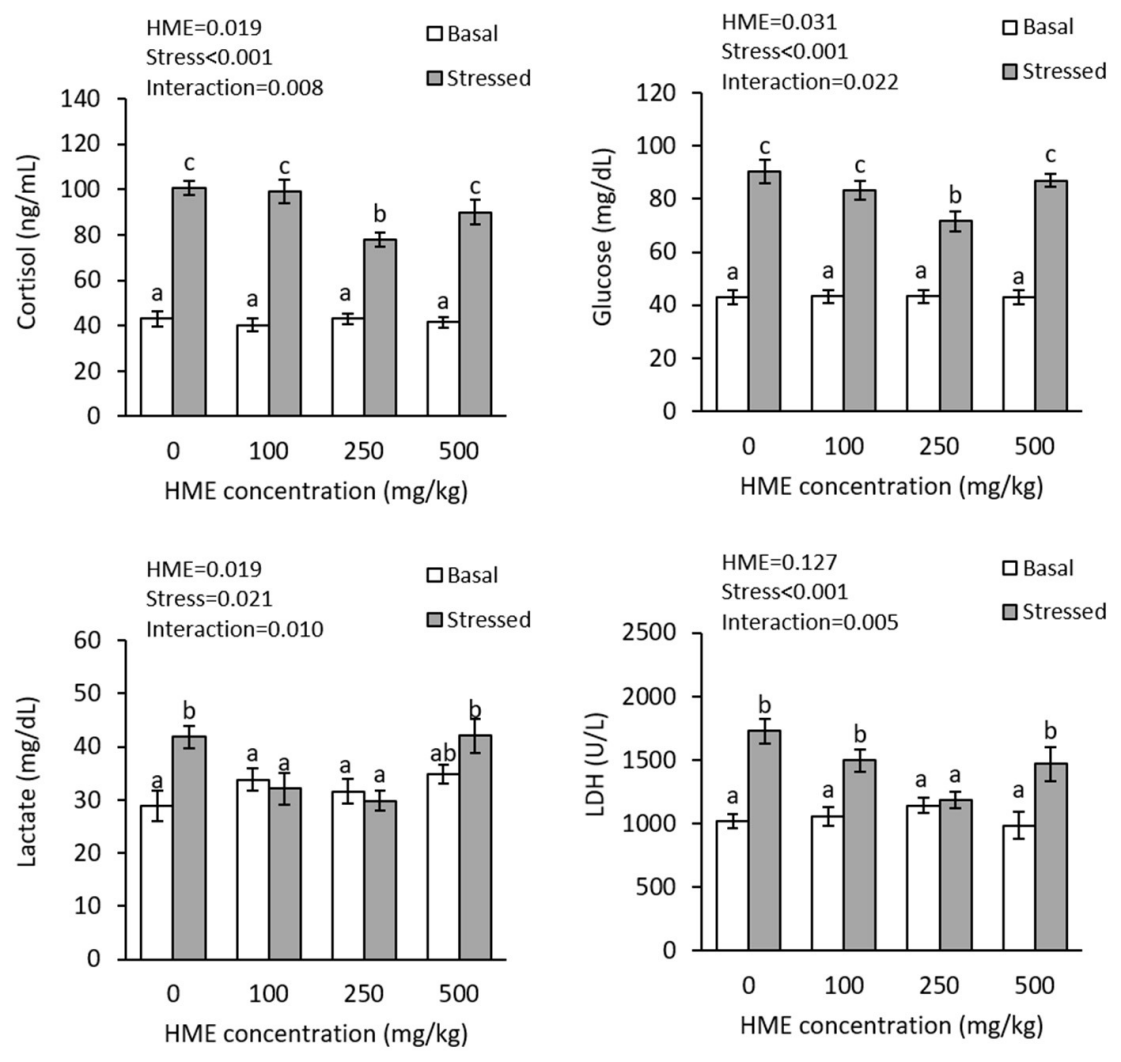
Plasma cortisol, glucose, lactate, and LDH (mean ± SE) levels in rainbow trout fed diets continuing 0–500 mg/kg HME for 63 days and subsequently exposed to thermal stress (16–22°C) for 7 days. Different letters above the bars indicate significant differences among the treatment combinations (dietary HME × stress; Duncan test; *n* = 6).

There were interaction effects of dietary HME and thermal stress on the plasma cortisol (*P* = 0.016), glucose (*P* = 0.007), lactate (*P* = 0.010), and LDH (*P* = 0.005). Thermal stress significantly increased the plasma cortisol levels. Dietary HME supplementation did not alter plasma cortisol, glucose, lactate, and LDH levels before thermal stress, but suppressed their elevation after thermal stress and the lowest levels were observed in 250E treatments for cortisol, glucose and LDH, as well as in 100E and 250E treatments for lactate (Figure 2).

Plasma AST activity was affected by thermal stress (*P* < 0.001), but not dietary HME (*P* = 0.106) and interaction of dietary HME and thermal stress (*P* = 0.343). After the thermal stress, all groups exhibited significant elevation in plasma AST activity (Table 3). Dietary HME (*P* < 0.001) and thermal stress (*P* < 0.001) had significant effects on plasma ALT activity; however there was no significant interaction effect (*P* = 0.326) of these factors on the enzyme activity. The fish in the 250E treatment exhibited significantly lower plasma ALT activity, compared to the control fish. Thermal stress led to significant elevation in the enzyme activity (Table 3). Plasma T4, and T3 levels were affected by dietary HME supplementation, but significantly decreased after thermal stress (*P* < 0.001). No significant interaction effects dietary HME and thermal stress have been observed on the plasma T4, and T3 levels (Table 3).

**Table 3:** Plasma ALT, AST, T4, and T3 (mean ± SE) levels in rainbow trout fed diets continuing 0–500 mg/kg HME for 63 days and subsequently exposed to thermal stress (16–22°C) for 7 days (*n* = 6).

	**HME (mg/kg)**	**ALT (U/L)**	**AST (U/L)**	**T4 (μg/mL)**	**T3 (ng/mL)**
Basal	0	23.1 ± 2.72	242 ± 15.9	16.3 ± 0.54	8.32 ± 0.51
	100	16.5 ± 1.60	239 ± 18.4	16.1 ± 0.53	7.82 ± 0.50
	250	12.3 ± 1.36	172 ± 9.94	17.8 ± 0.64	7.97 ± 0.61
	500	15.2 ± 2.00	233 ± 16.7	17.1 ± 0.63	8.32 ± 0.59
Stressed	0	54.8 ± 3.50	564 ± 23.6	11.6 ± 0.34	2.63 ± 0.27
	100	46.6 ± 2.21	559 ± 28.0	11.6 ± 0.30	2.88 ± 0.24
	250	36.6 ± 2.32	543 ± 24.3	12.1 ± 0.35	2.90 ± 0.31
	500	40.4 ± 2.42	534 ± 14.7	12.4 ± 0.42	2.62 ± 0.31
P-value					
HME		0.001 > 0^b^, 100^ab^, 250^a^, 500^ab^	0.106	0.075	0.992
Stress		0.001>; Basal < Stressed	0.001>; Basal < Stressed	0.001>; Basal > Stressed	0.001>; Basal > Stressed
Interaction		0.326	0.343	0.577	0.741

The superscript letters indicate the significant differences among the HME concentrations.

**Figure 3:**
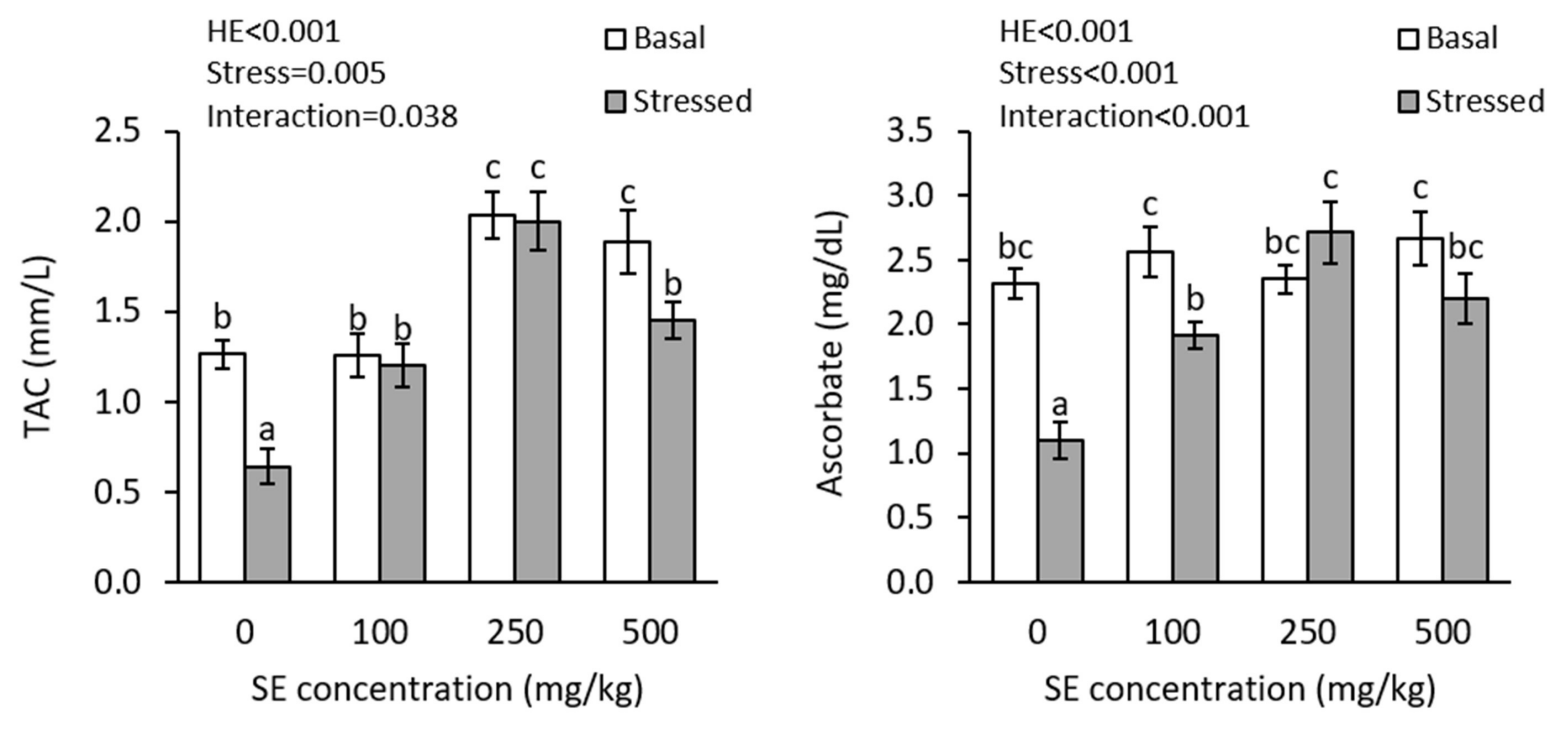
Plasma TAC and ascorbate (mean ± SE) levels in rainbow trout fed diets continuing 0–500 mg/kg HME for 63 days and subsequently exposed to thermal stress (16–22°C) for 7 days. Different letters above the bars indicate significant differences among the treatment combinations (dietary HME × stress; Duncan test; *n* = 6).

There were interaction effects of dietary HME and thermal stress on plasma TAC (*P* = 0.038) and ascorbate (*P* < 0.001) levels (Figure 3). TAC significantly increased in both 250E and 500E treatments, before thermal stress; whereas, plasma ascorbate showed no significant changes in control, 100E, 250E, and 500E treatments at this time. Thermal stress led to significant decline in plasma TAC in control and 500E treatments. After thermal stress, the highest plasma TAC level was observed in 250E, whereas the lowest level was observed in the control treatment. Plasma ascorbate levels significantly decreased in the control and 100E treatments, after thermal stress. The highest plasma ascorbate level at this time was observed in 250E treatment.

**Figure 4:**
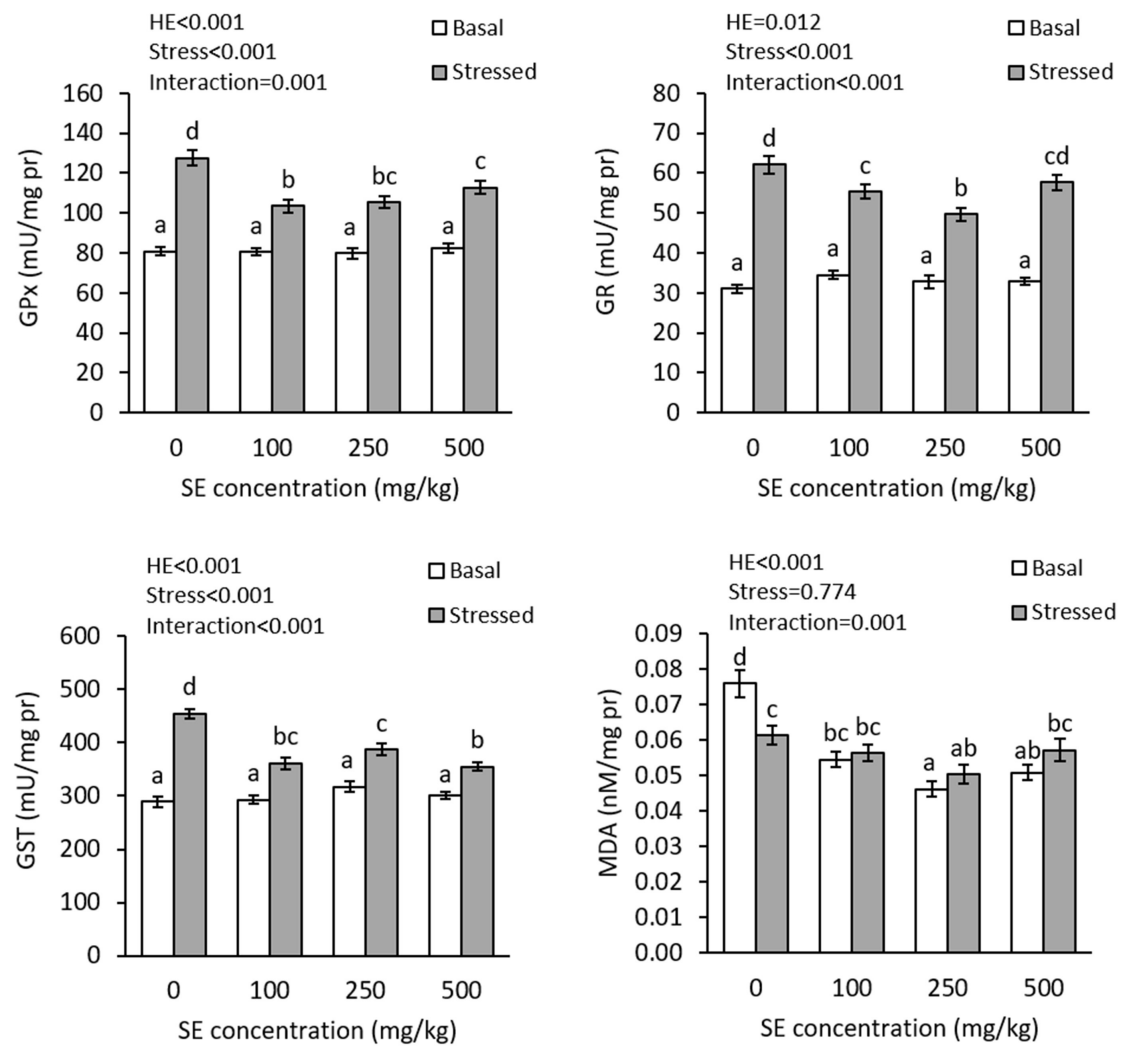
Gill GPx, GR, GST, and MDA (mean ± SE) in rainbow trout fed diets continuing 0–500 mg/kg HME for 63 days and subsequently exposed to thermal stress (16–22°C) for 7 days. Different letters above the bars indicate significant differences among the treatment combinations (dietary HME × stress; Duncan test; *n* = 6).

The results showed interaction effects of dietary HME and thermal stress on the gill GPx (*P* = 0.001), GR (*P* < 0.001), GST (*P* < 0.001), and MDA (*P* = 0.001) (Figure 4). There were no significant differences in the gill GPx, GR, and GST activities before thermal stress; nevertheless, HME-treated fish showed significantly lower gill MDA levels, compared to the control treatment and the lowest level was observed in the 250E and 500E treatments. After thermal stress, there were significant elevations in the gill GPx, GR, and GST activities in all treatments. At this time, 100E and 250E exhibited lower gill GPx, GR, and GST elevation, whereas 500E showed lower gill GPx and GST activities, compared to the control treatment. The lowest GPx, GR, and GST activities were observed in 100E, 250E, and 500E treatments, respectively. Thermal stress significantly decreased the gill MDA level in the control fish, but induced no changes in the other treatments.

## Discussion

Herbal additives are best known for their antioxidant and immunostimulant properties; however, there are several reports about the growth-promoting characteristics of herbal additives, which may be related to enhanced digestion and/or absorption (36–38). Such a growth promotion must be carefully interpreted as some studies have reported high weight gain with no change in feed efficiency, when fish were fed herbal additive-supplemented diet, indicating higher feed intake because of dietary supplementation (39–42). Based on the present results, it is speculated that HME may have no benefits on nutrient digestion and/or absorption, which are similar to reports of supplementing diets with Russian olive, *Elaeagnus angustifolia*, leaf extract in common carp (43), ginkgo, *Ginkgo biloba*, leaf extract in hybrid grouper (*Epinephelus lanceolatus*♂ × *Epinephelus fuscoguttatus*♀) (44), and Chinese ginseng, *Panax ginseng*, extract in rainbow trout (45).

The present study demonstrated that thermal stress induced physiological stress in the fish, characterized by elevations in plasma cortisol, glucose, lactate and LDH. These results are in line with previous findings in rainbow trout (20) as well as other fish species (46–49). The overall consequences of such changes in plasma parameters are an increase in energy supply to cope with the thermal stress-induced negative effects. However, these responses have side effects such as shifted energy allocation toward catabolism processes, immunosuppression, osmoregulation disturbance, and behavioral alterations. Consequently, various strategies including nutritional modulation for thermal stress suppression are desirable requirements in culture practices. There are a few studies focusing on the role of dietary herbal additives on fish stress response during exposure to high water temperature. A mitigation in cortisol elevation has been observed in thermally stressed Nile tilapia, when rocket, *Eruca sativa* (50), or lion's mane (27) leaves' powder was added to the fish diet. According to the present results, HME at 250E was capable to suppress thermal stress. Whether such an anti-stress effects of HME relates to direct effect on the hypothalamic- pituitary-interrenal axis or is the result of “boosting” the fish overall welfare warrants further research.

Thyroid hormones have a wide-ranging role in fish physiology such as involvement in fish growth and anabolic metabolism, osmoregulation, and reproduction (51). It has been proposed that the hypothalamic-pituitary-thyroid axis interact with the hypothalamic-pituitary-interrenal axis in fish (52). Thyrotropin-releasing hormone is known to stimulate cortisol release under stress, beside its roles in the thyroid axis (53). However, several studies on fish have shown that blood thyroid hormone levels decrease under stressful conditions, such as thermal stress (6, 48, 54). The mechanisms behind such decrements in the hormonal levels are not proposed yet, but fish growth depression and hydromineral imbalance under heat stress may be partially due to this change. According to the present findings, although HME suppresses thermal stress in the fish, it fails to restore thyroid hormonal level in rainbow trout.

One of the negative consequences of thermal stress in fish is hepatic damage, which may be due to accelerated metabolism and production of reactive oxygen species and free radicals (5, 6). Plasma ALT and AST activities have been found elevated in fish, following exposure to high water temperature (6–8) and the present results are in agreement with these studies. Herbal additives may act as hepatoprotective agents, potentially because of their antioxidant and detoxifying traits (55). The lower plasma ALT activity in 250E treatment may suggest higher hepatic health in this group of fish. There is no report on hepatoprotective characteristics of Hyssop and this topic is worth conducting further researches.

Occurrence of oxidative stress is one of the main concerns associated with thermal stress in fish. An increase in water temperature elevates respiration and metabolism rate, leading to elevated formation of reactive oxygen species (2). This leads to a higher risk of oxidative stress in fish with many physiological and metabolic consequences. The present results clearly indicate that the thermal stress resulted in oxidative conditions in the fish. Oxidative conditions lead to depletion of reductive compounds in the fish blood, which was characterized by lowering of plasma TAC and ascorbate levels in the present study. Role of ascorbic acid in thermal stress resistance has been proposed in fish (56, 57) and it has been reported that thermal stress results in the diminishing of tissue ascorbate concentration and MDA elevation in freshwater catfish (58). Moreover, TAC is a measure of all reducing compounds in the fish blood and exhibits decrements, when fish are under oxidative conditions (59). It has been reported that thermal stress causes decrease in plasma and muscle of matrinxã, *Brycon amazonicus*, along with an increase in lipid peroxidation (60). Therefore, the present study indicates that dietary HME is capable to suppress oxidative conditions in trout, during thermal stress exposure. Such an improvement in thermal tolerance may be due to augmenting antioxidant capacity before the stress event (increase in TAC in 250 and 500 mg/kg HME) that provides an adequate source of defensive compounds to combat the oxidative conditions after the stress.

The fish gill is in direct contact to the surrounding water and water temperature elevation affects this exposed organ in many ways. Although HME presented no significant improvement in the gill antioxidant enzymes' activities, it significantly suppressed MDA content, suggesting lower lipid peroxidation in the organ. This may be an indicator of the radical-scavenging property of the extract that suppressed lipid peroxidation without activation of the enzymatic antioxidant system. Several studies have reported radical-scavenging activity of different herbal materials (61). Moreover, radical-scavenging characteristics of Hyssop have been confirmed, *in vitro* (31). Despite the results of plasma TAC and ascorbate levels, the gill MDA values suggests that thermal stress induced no lipid peroxidation in the fish. This may be due to an adaptation in the fish that decreased the proportion of unsaturated fatty acids in the gills. Studies have shown that unsaturated fatty acid proportion of lipids decrease in tissues of fish, when the fish are maintained at higher temperature and cell membrane re-modeling occurs (62, 63). Under this situation, substrates for lipid peroxidation decreases, which mitigates the thermal-induced oxidative stress. However, the gill GPx and GR elevations suggest an elevation in hydrogen peroxide and organic hydroperoxide detoxification (64). On the other hand, GST is responsible for xenobiotic detoxification and the elevation in the enzyme activity in the fish gill suggest that the thermal stress has induced oxidative stress, but not exclusively in fatty acids according to Tsuchida (65). Overall, the present results are in line with previous studies, showing dietary herbal additives can mitigate oxidative conditions caused by thermal stress in fish. For example, application of Chinese rhubarb extract in giant freshwater prawn (24), *Laminaria* sp. in Atlantic salmon (26), and lion's mane and oregano essential oil in Nile tilapia (25, 27) have all been reported to significantly mitigate oxidative stress caused by thermal stresses.

It is concluded that dietary HME, although having no direct benefits on rainbow trout growth performance, has nonetheless measurable health attributes such as hepatoprotection and improvement in specific antioxidative responses. Moreover, a period of dietary HME supplementation is beneficial in reducing adverse effects of temperature elevation such as stress responses, hepatic damage, and oxidative stress. Based on the results, 250 mg/kg HME is the optimum concentration for rainbow trout feed supplementation in practice.

## Data availability statement

The original contributions presented in the study are included in the article/Supplementary materials, further inquiries can be directed to the corresponding authors.

## Ethics statement

The studies involving human participants were reviewed and approved by Ministry of Defense Research Ethics Committee (MODREC). All participants provided written informed consent.

## Author contributions

MY, SH, EK, and SS: conceptualization. EK, SS, AP, and NB: study design. AP and NB: data analysis. MK, MY, and SH: methodology. SD and SH: drafting. SH and MY: supervision. MY: grant acquisition and editing. All authors contributed to the article and approved the submitted version.

## Funding

This publication has been supported by the RUDN University Scientific Projects Grant System, Project No. 202196-2-174.

## Conflict of interest

The authors declare that the research was conducted in the absence of any commercial or financial relationships that could be construed as a potential conflict of interest.

## Publisher's note

All claims expressed in this article are solely those of the authors and do not necessarily represent those of their affiliated organizations, or those of the publisher, the editors and the reviewers. Any product that may be evaluated in this article, or claim that may be made by its manufacturer, is not guaranteed or endorsed by the publisher.
